# Palliative care knowledge among nursing students at a Polish medical university: a cross-sectional study

**DOI:** 10.3389/fmed.2026.1873987

**Published:** 2026-09-23

**Authors:** Natalia Pisarek, Tomasz Kryczka

**Affiliations:** Department of Development of Nursing and Social & Medical Sciences, Medical University of Warsaw, Warsaw, Poland

**Keywords:** knowledge, long-term care, nurse, nursing students, palliative care

## Abstract

**Introduction:**

Non-communicable diseases, such as neurological disorders manifesting as dementia, often necessitate a shift in the nature of care – from long-term to palliative. Nurses play a particularly important role in this transition, as they spend the most time with patients: both in healthcare facilities and in home-care settings. Their competencies begin to develop already during their medical education. Therefore, the aim of this study was to assess nursing students’ knowledge of palliative care.

**Methods:**

The university-based cross-sectional study was conducted among nursing students (*n* = 925) from one of the oldest and leading universities in Poland. The study was based on a survey questionnaire consisted of a demographic part, Palliative Care Knowledge (PaCKs) scale, and 17 closed-ended, single-choice questions evaluating the students’ detailed knowledge of palliative care. Statistical significance (*p* < 0.05) was determined using the Chi-square test.

**Results:**

Polish nursing students (*n* = 925; average response rate, 83%) demonstrate a moderate level of knowledge regarding palliative care, as reflected in both the 13-item PaCKs scale score (9.64 ± 1.99; mean ± SD) and the percentage of correct answers to 17 researcher-developed questions (53.25%). Associations were identified between correct answers on the PaCKs scale and the age of respondents (7 items; *p* = 0.0024–0.0438), year of study (6 items; *p* = 0.001–0.0499), and place of residence (2 items; *p* = 0.0121 and 0.0266). Only one-fifth of respondents had ever considered pursuing a career in palliative care after graduation.

**Conclusions:**

Although the results of the study were relatively satisfactory, it is evident that nursing students require greater clarity and a deeper understanding of the nuances of palliative care. Addressing this knowledge gap may be achieved through comprehensive education that covers palliative, hospice, and long-term care, clarifying the appropriate contexts for each. Some of the more unexpected observations warrant further investigation.

## 1 Introduction

Scientific advancements and improvements in life expectancy have contributed to population aging. It is estimated that by 2060 the number of individuals experiencing serious health-related suffering at the end of life will increase by 87%, reflecting an epidemiological shift towards a higher prevalence of incurable life-limiting diseases (1). Non-communicable diseases (NCDs) represent an important category within thig group, and more specifically, a significant proportion of these diseases comprise neurological disorders leading to dementia (2, 3). Such patients require access to long-term care. In certain clinical scenarios, such care provided by family members or professional caregivers or nurses, turns to palliative care (PC). There is also a whole group of diseases in which PC may be introduced early and administered alongside disease-modifying treatment [e.g., (4–6)].

Over the last two decades, the World Health Organization (WHO) has advocated for PC to be considered as a public health priority, emphasizing the need for its earlier integration within broader healthcare system to improve access and availability. It is estimated that approximately 40 million people worldwide need PC, yet only about 14% are currently receiving it. Although PC is not a novel concept, access to it remains inadequate and neglected in developing countries. In fact, international research suggests that PC is poorly understood among the general public (7).

Meanwhile, high-quality PC can be ensured by a multidisciplinary team led by nurses, who have more frequent contact with patients than other healthcare professionals. Given their greater presence at the patient's bedside compared to other healthcare professionals, they should be considered the core of such teams, with their primary responsibilities encompassing the coordination of all specialists, integration of all clinical recommendations, and individualisation of care to a particular patient (8).

Nevertheless, PC represents a challenging domain of patient care, requiring comprehensive preparation encompassing practical, scientific, spiritual, and communication competencies, both verbal and nonverbal. Consequently, additional specialized training beyond undergraduate education is considered essential. The foundation of such preparation lies in the knowledge acquired during undergraduate studies and delivered across various courses at different levels of education. Therefore, this study aimed to assess nursing students’ knowledge of palliative care at each year of their studies.

## 2 Materials and methods

### 2.1 Study setting and sampling

The study population comprised nursing students of the Medical University of Warsaw in Poland, representing all study years of BSc and MSc programmes. The Medical University of Warsaw was selected for this study as it is recognized as one of the leading medical universities in the country, serving as a reference institution in the context of the quality of education.

Participation in the study was voluntary and anonymous. Participants were informed about the study's purpose and design, following which written consent to participate in the study was obtained. Participants who did not provide consent were excluded from the study. Students could withdraw from the study at any time without obligation to provide a reason. In the event of withdrawal, the completed questionnaire was destroyed by the supervising researcher.

The study was conducted using a dual-track approach: data were collected from students via paper-based questionnaires and via an online questionnaire distributed through the university's internal network. Data collection via paper-based questionnaires was coordinated with university lecturers, with measures taken to minimize any interference with daily university routine or professional responsibilities. The number of questionnaires from each student group was counted and checked for completeness of responses. Incomplete questionnaires were rejected. This allowed us to determine the percentage of students who completed the questionnaire and the percentage of incorrectly completed forms. For the online form, the link to the questionnaire was sent to student groups that had not completed the questionnaire during teaching sessions. The percentage of completed questionnaires was calculated based on the number of questionnaires received and the number of students in the group. Online questionnaires that were not completed in full were counted and excluded from further analysis. The number of respondents for each year group was totalled and compared with the total number of students in that year group, thereby yielding the response rate. The minimum sample size was calculated considering the size of the target population (1,115 students), a confidence level of 95%, expected prevalence of 80% and precision of 0.05, and was found to be 286.

### 2.2 Survey

The study employed a Polish-language survey questionnaire comprising a demographic section (gender, age, education/year of study, place of residence), including participants’ experience in PC, the Palliative Care Knowledge Scale (PaCKs), and 17 closed-ended, single-choice questions evaluating the students’ detailed knowledge of PC. The content of the questions was developed on the basis of existing publications on palliative care [e.g., (9)], in collaboration with academic staff who teach PC, healthcare professionals involved in PC, and examiners responsible for preparing questions for the specialisation examination in palliative medicine. Although the questions were prepared in collaboration with linguists and palliative care specialists, they did not undergo formal psychometric validation.

The Palliative Care Knowledge Scale used in this study is a validated tool for assessing preliminary knowledge of PC. It contains 13 items addressing various aspects of PC, each answered via ‘True’ or ‘False’ responses. To avoid random guessing, a third option, “I do not know.”, was included. This modified version was adapted from a study done by Kozlov et al. (10). Responses on the PaCKs scale were scored by summing correct answers. Each correct answer was awarded 1 point, while incorrect answers and ‘don't know’ responses were awarded 0 points. The score range was 0–13, and a non-linear classification of results was adopted: low knowledge (0–6), moderate knowledge (7–10), high knowledge (11–13). The Polish version of PACKs was translated by language experts and verified for linguistic accuracy under the supervision of specialists in computer science and sociology.

### 2.3 Inclusion and exclusion criteria

Eligibility criteria required participants to be aged 18 years or older, capable of providing informed consent, and affiliated with Medical University of Warsaw as nursing students. Additionally, all participants were required to demonstrate sufficient Polish language proficiency to understand and complete the questionnaire.

Exclusion criteria encompassed individuals under the age of 18 years, those unable to provide informed consent, nursing students not affiliated with Medical University of Warsaw, first-year students, and individuals with a self-declared lack of willingness to complete the questionnaire.

### 2.4 Data analysis

All statistical calculations were performed using STATISTICA 12.0 statistical package (StatSoft. Inc., USA). Quantitative variables were characterized using the arithmetic mean, standard deviation, median, minimum and maximum values and 95%CI. Qualitative variables were presented using counts and percentages.

Chi-square tests of independence were used for qualitative variables (with the use of Yates’ correction for cell counts below 10, Cochran’s *Q* test, Fisher’s exact test, as appropriate).

### 2.5 Ethical considerations

The study was conducted in accordance with the principles of the Declaration of Helsinki. Participation in the questionnaire was voluntary, and participants had the right to withdraw at any time. All responses were kept strictly confidential, ensuring full anonymity for the participants. Informed consent was obtained from all participants prior to their inclusion in the study. The study was approved by the Bioethics Committee of the Medical University of Warsaw (Approval No. AKBE/46/2023).

## 3 Results

### 3.1 Study group

Given the number of students at the Medical University of Warsaw (WUM), a threshold of over 50% was set as the minimum sample size. The study involved 1115 students. Incorrectly completed questionnaires were rejected from further analysis. In practice, nearly 83% (*n* = 925; Female/Male: 856/69) of all students surveyed (Table 1) and the number of completed paper-based surveys was 253. The response rate for the individual years of study was as follows: 1. BSc 61.4%, 2. BSc 90.7%, 3. BSc 95.9%, 1. MSc. 97.2% and 2. MSc 96.5%. Majority of the students were <22 years old (51.4%) and in their first or second year of BSc study (25.2% and 28.4% of all respondents, respectively; Table 1).

**Table 1:** Study group characteristics.

Variables	Respondents (*n* = 925)
Gender^a^	
Female	92.5%
Male	7.5%
Age	
≤20 years	25.7%
21 years	25.7%
22 years	14.2%
23 years	15.0%
≥24 years	19.4%
Place of living	
Village	21.3%
Town < do 50,000 inhabitants	13.8%
Town 50–150,000 inhabitants	12.6%
Town 150–500,000 inhabitants	24.7%
City >500,000 inhabitants	27.4%
Year of the study	
1. BSc (*n* = 234; 61.4%)^b^	25.2%
2. BSc (*n* = 263; 90.7%)^b^	28.4%
3. BSc (*n* = 211; 95.9%)^b^	22.8%
1. MSc (*n* = 105; 97.2%)^b^	11.3%
2. MSc (*n* = 112; 96.5%)^b^	12.1%
Have you ever heard of palliative care?	
Yes	99.2%
No	0.8%
Has anyone in your family or friends needed or had access to palliative care services?	
Yes	30.4%
No	69.6%
Have you cared for someone at the end of their life?	
Yes	51.8%
No	48.2%
Have you ever thought about working in palliative care?	
Yes	19.8%
No	80.2%

BSc, bachelor degree studies; MSc, master degree studies.

a self-reported.

b values in brackets: (number of respondents; response rate).

The vast majority of students (99.2%) reported having heard of PC, although only 30.4% indicated having close relatives who needed access to such services. More than half of respondents (51.8%) reported having provided care for a terminally ill individual; however, only 19.8% expressed an interest in pursuing a career in PC (Table 1).

### 3.2 Findings

#### 3.2.1 Palliative care knowledge scale

Students responded to 13 statements addressing various aspects of PC using ‘True’, ‘False‘ or ‘I do not know’ response options. The proportion of correct answers ranged from 43.9% to 92.9% (Cronbach's alpha = 0.86). The highest proportion of correct answers were recorded for statements that ‘palliative care helps the whole family to cope with a serious illness’ and ‘palliative care is a team-based approach to care’ (92.9% and 92.5%, items 13 and 9, respectively; Table 2). The lowest proportions of correct responses were recorded for items 6 and 10, (both 43.9%; Table 2).

**Table 2:** PaCKs questionnaire responses.

Questions	Correct	Incorrect	Don't know
Item 1: One goal of palliative care is to address any psychological issues brought up by serious illness. (T)^b^	64.0%	25.7%	10.3%
Item 2 Stress from serious illness can be addressed by palliative care. (T)^a^^,^^c^^,^^d^	48.2%	31.6%	20.2%
Item 3: Palliative care can help people manage the side effects of their medical treatment. (T)^a^^,^^c^	79.1%	11.1%	9.9%
Item 4: When people receive palliative care, they must give up their other doctors. (F)	89.7%	4.0%	6.3%
Item 5: Palliative care is exclusively for people who are in the last six month of life. (F)^a^^,^^c^	61.3%	13.4%	25.3%
Item 6: Palliative care is specifically for people with cancer. (F)	43.9%	50.6%	5.5%
Item 7: People must be in the hospital to receive palliative care. (F)^c^	90.1%	3.6%	6.3%
Item 8: Palliative care is designed specifically for older adults. (F)^a^^,^^c^^,^^d^	68.0%	29.6%	2.4%
Item 9: Palliative care is a team-based approach to care. (T)	92.5%	2.0%	5.5%
Item 10: One goal of palliative care is to help people better understand their treatment options. (T)^a^	43.9%	39.5%	16.6%
Item 11: Palliative care encourages people to stop treatments aimed at curing their illness. (F)^a^	84.2%	6.7%	9.1%
Item 12: One goal of palliative care is to improve a person's ability to participate in daily activities. (T)^a^^,^^b^^,^^c^	75.1%	15.0%	9.9%
Item 13: Palliative care helps the whole family cope with a serious illness. (T)	92.9%	4.7%	2.4%

Statistical significance (*p* < 0.05) was determined using the Chi-square tests.

*n* = 925; T, true; F, false; *p* < 0.05, significance of correct answers in relation to:

a age.

b place of residence.

c year of study.

d experience in caring for terminally ill patient.

Correct responses to the majority of items included in the PaCKs scale (items 2, 3, 5, 8, 10, 11, and 12; Table 2) were positively associated with respondents’ age (*p* < 0.05), with the strongest association observed between age and the correct response to the statement ‘palliative care is designed for older adults’ (item 8, Table 2; *p* = 0.0002). An exception was noted for item 11 (Table 2), where 22-year-old students significantly outperformed other age groups in the frequency of correct responses (score:100%, *p* = 0.0122).

Although PaCKs scale scores were generally positively associated with the year of study the significant differences were noted for items 2, 3, 5, 7, 8, 12 (Table 2, *p* < 0.05). First-year MSc students demonstrated a higher proportion of correct responses than those in their second year.

Place of residence was found to influence responses only for items 1 and 12 (Table 2), with correct response rates increasing in proportion to the population size of the students’ permanent residence (*p* = 0.0121 and *p* = 0.0266).

A significant association between experience in caring for the terminally ill and correct response rates was limited to items 2 and 8 (Table 2) of the PaCKs scale (*p* = 0.0262 and *p* = 0.0032, respectively).

Table 3 presents PaCKs scores stratified by demographic variables. No significant differences were noted.

**Table 3:** PaCKs scores across variables (*n* = 925).

Variables	Score (Mean ± SD)
Gender:	
Female	9.29 ± 2.27
Male	9.74 ± 1.63
Age:	
≤20 years	8.98 ± 1.96
21 years	8.48 ± 2.29
22 years	9.83 ± 2.12
23 years	9.84 ± 2.09
≥24 years	10.41 ± 2.14
Place of living:	
Village	9.5 ± 2.26
<50,000 inhabitants	9.57 ± 2.33
50,000 to 150,000 inhabitants	8.31 ± 2.40
150,000 to 500,000 inhabitants	9.00 ± 2.04
>500,000 inhabitants	9.48 ± 2.11
Year of study:	
BSc program – 1st year	8.28 ± 2.19
BSc program – 2nd year	8.84 ± 2.12
BSc program – 3rd year	12.00 ± 1.41
MSc program – 1st year	10.11 ± 2.20
MSc program – 2nd year	9.65 ± 2.23
Have you heard of palliative care before?	
Yes	9.34 ± 2.23
No	8.00 ± 2.83
Have you or any of your close relatives needed or had access to palliative care?	
Yes	9.47 ± 2.07
No	9.27 ± 2.30
Have you taken care of someone at the end of life?	
Yes	9.58 ± 2.07
No	9.06 ± 2.37
Have you ever considered working in palliative care?	
Yes	9.66 ± 2.01
No	9.25 ± 2.28

#### 3.2.2 Detailed knowledge about palliative care

The detailed section (Table 4) focused on fundamental medical procedures, pharmacological agents, and their respective routes of administration. The proportion of correct responses ranged from 4.3% to 92.5%. Correct response rates were notably lowest for items 5 and 14, which evaluated the management of appetite loss in terminally ill patients (4.5% and 11.1%, respectively; Table 4). Age was positively associated with correct responses for items 4, 9, 10, 11, 15, 16 and 17 (Table 4). Item 12 deviated from this trend, with students aged 22 years and older demonstrating the highest frequency of correct responses (Table 4).

**Table 4:** Detailed knowledge about palliative care.

Questions	Responses (*n* = 925)
Item 1: A characteristic feature of breakthrough pain is:	
Long duration	15.0%
Rapid onset (T)	55.7%
Well tolerated by patients	0.4%
Occurs before the end of the dosing interval	16.2%
I don't know	12.6%
Item 2: A nurse measures pain intensity using:	
The VAS (visual analogue scale)	1.6%
The NRS (numerical rating scale)	5.1%
Observation (e.g., facial expressions, gestures)	0.4%
All of the above (T)	92.5%
I don't know	0.4%
Item 3: When inserting a subcutaneous ‘butterfly needle’ into the upper arm or thigh:	
The needle should be directed upwards (T)	49.0%
The needle should be directed downwards	20.2%
The needle should be directed inward	13.4%
Monitor basic vital signs	0.4%
I don't know	17.0%
Item 4: The preferred route for administering an analgesic is:	
Intramuscular	5.5%
Oral (T)^a^^,^^b^	26.9%
Intravenous	55.7%
Rectal	1.6%
I don't know	10.3%
Item 5: Terminally ill patients may experience weakness, loss of appetite, and changes in the taste and smell of food. Nursing management should consist of:	
Providing frequent, small meals (T)^b^	4.3%
Monitoring the amount of carbohydrates consumed	4.7%
Providing meals high in fibre	1.2%
Correct answers a. and b.	87.0%
I don't know	2.8%
Item 6: Psychogenic vomiting in cancer patients may be caused by:	
Previous experiences associated with chemotherapy (T)^c^	67.2%
Increased intracranial pressure	6.7%
Changes in the gastrointestinal tract	9.1%
Gastritis or gastroenteritis	6.3%
I don't know	15.0%
Item 7: In terminally ill patients, one of the main symptoms is chronic fatigue syndrome, most commonly manifested by:	
physiological complaints, e.g., urinationi/physiological complaints, e.g., urination disorders	0.8%
Mental complaints, e.g., personality disorders	0.8%
Both somatic and mental complaints, e.g., lack of motivation, insomnia, general discouragement (T)	84.2%
Somatic symptoms, e.g., excessive sleepiness, headaches	11.9%
I don't know	2.4%
Item 8: Acceptance of the appropriate time of death means:	
Neither hastening nor delaying the moment of death (T)	87.4%
Taking actions to shorten the patient's suffering	6.7%
Accepting the patient's request for euthanasia	2.0%
Taking all possible actions to prolong life	2.4%
I don't know	1.6%
Item 9: A narcotic analgesic available in transdermal form is:	
Fentanyl	23.0%
Buprenorphine	7.5%
Methadone	2.4%
Correct answers a. and b. (T)^a^^,^^d^	59.1%
I don't know	7.9%
Item 10: The term “asthenia” refers to:	
Difficulty urinating, fatigue	2.0%
Hair loss, tiredness, dry mouth	11.5%
Fatigue, tiredness, exhaustion (T)^a^^,^^b^	39.7%
Fatigue, tiredness, difficulty urinating	6.0%
I don't know	40.9%
Item 11: In xerostomia caused by chemo- and radiotherapy, relief is brought to the patient by:	
Drinking cola-type beverages	3.2%
Eating raw fruits and vegetables	0.4%
Sucking ice cubes, sucking candies, or chewing gum (T)^a^^,^^b^^,^^c^	40.1%
All of the above	20.6%
I don't know	35.7%
Item 12: Which factors increase the pain threshold?	
Symptom relief, anxiety, sleeping	5.2%
Understanding, presence of others, sadness	4.8%
Internal isolation, understanding, sleeping	2.8%
Rest, sleeping, distraction (T)^a^^,^^b^^,^^d^	79.4%
I don't know	7.9%
Item 13: The most common cause of chronic fatigue syndrome in cancer patients is:	
Anaemia (T)	38.9%
Polycythaemia	1.6%
Dehydration	28.2%
Hypovolaemia	10.3%
I don't know	21.0%
Item 14: Megestrol acetate is used in patients to:	
Prevent vomiting	7.9%
Improve appetite (T)	11.1%
Prevent diarrhoea	4.4%
All of the above	25.4%
I don't know	51.2%
Item 15: In cancer cachexia, meals should be rich in:	
Protein (T)^a^^,^^c^	75.4%
Vitamins and minerals	12.3%
Fats and vitamins	6.7%
Potassium and calcium	2.4%
I don't know	3.2%
Item 16: On the second step of the ‘WHO analgesic ladder’, the following are used:	
Only non-opioid analgesics	6.0%
Strong opioids + co-analgesics	3.6%
Weak/mild opioids	14.3%
Weak/mild opioids + non-opioid analgesics (T)^a^^,^^b^	69.4%
I don't know	6.7%
Item 17: Which of the following is NOT an indication for palliative care in adults?	
Cancer	4.8%
Pressure ulcers (decubitus ulcers)	35.7%
AIDS	21.8%
Right ventricular heart failure (T)^a^^,^^b^^,^^c^^,^^d^^,^^e^	25.0%
I don't know	12.7%

Statistical significance (*p* < 0.05) was determined using the Chi-square tests.

T, true; *p* < 0.05, significance of correct answers in relation to:

a age.

b year of study.

c experience in caring for terminally ill patient.

d palliative care service identified in relatives or friends.

e positive attitude towards working in palliative care.

The year of study was positively associated with correct responses for items 4, 5, 10, 11, 12 and 17, whereas a negative correlation was observed for items 15 and 16 (Table 4).

Prior exposure to PC through a family member or close relative was found to be significantly positively associated with correct responses to items 9, 12 and 17 (*p* = 0.03, *p* = 0.04, *p* = 0.03, respectively; Table 4). Furthermore, experience in providing care for terminally ill patients was significantly associated with higher correct response rates for items 6, 11 and 17 (*p* = 0.03, *p* = 0.02, *p* = 0.002, respectively; Table 4). An expressed interest in a PC career was positively associated with correct responses for item 17 only (*p* = 0.0044; Table 4).

## 4 Discussion

Common misconceptions include a tendency to associate PC exclusively with patients diagnosed with cancer. As a result, non-oncological patients are often excluded from PC, and family members of individuals living with chronic illnesses may assume that this service is not suitable for their loved ones (11). Unfortunately, this is a widespread belief in many societies; moreover, there are countries where most people have not even heard of PC, and many physicians associate it only with pain management and restrict its use to terminally ill patients. Due to this misconception, physicians often fail to identify many patients who need PC but do not fall into any of the previously mentioned groups. This limits access to these services (12–15).

In reality, PC is a component of long-term care, where clinical management is less challenging than in situations involving patients with extremely limited self-care capacity during the terminal phase of illness. Conversely, in some conditions there may be no transition from long-term care to palliative care (PC), as elements of palliative care (PC) are required from the very outset. Knowledge in this area appears crucial given the ageing of populations worldwide and the rather fluid boundary between long-term care and PC, especially in non-communicable diseases (NCDs) such as neurological, respiratory, or cardiovascular disorders, among others (2).

Because of the methodological differences between these two types of care, it is essential to identify the symptoms that are key to shifting the clinical approach taken by healthcare professionals. Nurses play a particularly important role here, as they spend the most time with patients: both in healthcare facilities and in home-care settings. In Poland, as in many other countries, the specialty of family and community nursing (16) operates within the system, with nurses forming part of the multidisciplinary primary-care team. It should be noted, however, that there is, despite family and community nursing also the specialty of palliative care nursing in Poland. PC nurses possess a high level of knowledge about PC and their experience, ongoing training, and positive attitudes contribute to improved care for palliative patients and their families (17).

These skills and competencies begin to be developed already during medical studies. In Poland, nursing studies are divided into two stages: three years leading to a bachelor's degree, followed by an additional two years to obtain a master's degree. Importantly, upon completing the bachelor's degree, nursing students also obtain the professional license required to practice as registered nurses (18). Consequently, pursuing further studies to obtain a master's degree is not obligatory for working directly with patients.

In our study, we assessed nursing students’ knowledge of PC, as reports from other countries indicate that medical students often have limited knowledge in this area (9, 19, 20). Meanwhile, Polish nursing students are familiar with the term ‘palliative care’, and nearly one-third reported that someone in their immediate environment (family or close relatives) had required such care. Half of the respondents had already cared for a person with an incurable illness (Table 1). This latter factor was significant in assessing students’ knowledge of PC using the PaCKS scale, where correct answers to two questions (items 2 and 8) were statistically significant in the context of such life experience (Table 2). It is also worth noting that despite a relatively high level of knowledge regarding PC – as indicated by the PaCKS scale scores of 9.29+/- 2.27 or 9.74+/−1.63 (F/M; mean+/-SD, Table 3) – and prior experience with terminally ill patients, only one-fifth of respondents had ever considered working in PC (Table 3). In our study, we did not attempt to explain students’ attitudes towards PC. However, we hypothesise (based on our conversations with students during classes) that presumably their understanding of this field, combined with the associated psychological or physical burdens, were key factors deterring them from pursuing this career path.

The knowledge level of nursing students, as measured by the PaCKs scale (Table 2), was consistent with data from countries that are culturally and organisationally distinct in terms of their healthcare systems (19–21), and was even higher than results reported in other European studies (22). Additionally, our study revealed noticeable differences in this knowledge (Table 3) favouring older or more educationally advanced students, which is unsurprising (21–24) as fragmented knowledge of PC is also acquired through various university courses.

Consequently, responses to certain questions on the PaCKS scale were significantly associated with the respondents’ age or year of study (Table 2). However, some answers were not as obvious to the students, particularly regarding questions that have also proven difficult in other studies (21). This applied to statements suggesting that PC is exclusively for people who are in the last six months of life (item 5, 61.3% correct), is intended specifically for people with terminal cancer (item 6, 43.9% correct), or that a goal of PC is to help people better understand their treatment options (item 10, 43.9% correct; Table 3). The fact that a substantial proportion of students struggled with these questions implies a degree of confusion between long-term, palliative, and hospice care, possibly stemming from the interchangeable use of these terms despite their distinct purposes and practices. A key issue is that this uncertainty may stem from the misconception that PC is exclusively reserved for end-of-life scenarios, where treatment options may be perceived as limited.

Regarding the PaCKS scale, correlations between the students’ place of residence and the number of correct answers for items 1 and 12 (concerning the goals of PC) were noteworthy. In both cases, the fewest correct answers came from students living in small towns of 50,000–150,000 inhabitants (Table 2). This is a rather surprising observation with no immediate justification, which likely warrants further investigation.

In terms of the PaCKs scale, it should be noted that, as expected, prior experience with PC among loved ones, direct care-giving experience, and a positive attitude toward working in the field translated into slightly higher mean values of knowledge compared to those who answered negatively. However, these differences were not statistically significant (Table 3).

The detailed section of the survey assessed knowledge regarding simple procedures and skills useful in PC. Correct responses ranged from 4.3% to 92.5%. The greatest challenge for students involved questions regarding the nutrition of patients with severe illness (items 5 and 14, with 4.3% and 11.1% correct answers, respectively; Table 4). It is possible, therefore, that this specific element of university education may require greater emphasis.

It is somewhat surprising that the optimal route of analgesic administration was incorrectly indicated by most respondents (26.9% correct answers), even though there is a positive correlation between the respondent's age or level of study advancement and answering this question correctly (item 4, Table 4). A similar pattern was observed for the questions on the definition of asthenia (item 10, Table 4), xerostomia (item 11, Table 4), factors that increase the pain threshold (item 12, Table 4), diet in cachectic conditions (item 15, Table 4) and the analgesic ladder (item 16, Table 4), and indications for palliative treatment (item 17, Table 4).

In our survey we wanted to find out how knowledgable students are about diseases that usually end in death and therefore require PC. For this reason, we compared ‘cancer’, ‘pressure ulcers’, ‘AIDS’ and ‘right ventricle heart failure’ (RVHF) in the question. The fact is that each of these ailments/diseases in an advanced stage requires PC. However, our intention was to determine the students’ reflexes in associating particular medical terms with terminal diseases and then – with PC. Among the diseases mentioned, only RVHF should be least associated with PC, especially since we did not use the term ‘advanced’ for this disease. To our disappointment, only one-quarter of respondents answered correctly (Table 4, item 17). This demonstrates a misunderstanding of the core philosophy of PC and a tendency to conflate PC with long-term care, a phenomenon that is, notably, not new [see (21)]. On the other hand, correct answers to this question were significantly more frequent among students who had personal contact with PC patients, had personally cared for such an individual, or were inclined toward a professional career in PC. Direct experience in caring for a terminally ill person, in particular, translated into significantly more frequent correct responses to the so-called practical items (items 6, 11, and 17, Table 4).

Previous studies show that nurses with a negative attitude toward PC tend to avoid caring for dying patients (24). Conversely, a positive attitude can enhance the quality of these services, whereas a negative one can hinder their provision (24–28). The willingness of nurses to engage in discussions about PC is also challenging: some believe that physicians hold the authority to make end-of-life decisions, while others report uncertainty when required to administer treatments prescribed by physicians, even when these conflict with their own beliefs. Studies show that nurses tend to avoid forms of care that evoke anxiety, helplessness, anger, distress, or fear of failure – a reaction that is entirely human and understandable (31, 30).

However, nurses currently constitute the largest group of healthcare professionals and play a central role in PC. They coordinate efforts between patients, their families, and other members of the healthcare team. Because they provide the vast majority of bedside care, it is essential that they possess adequate knowledge and training in PC to meet patient needs. Appropriate knowledge and training also support nurses in communicating effectively with patients and families and in alleviating their suffering (31, 32).

## 5 Limitations

This publication has several limitations. First, the study was conducted in a single university setting, and although it was carried out at one of the leading medical universities in Poland, its findings should be interpreted with caution when considering generalisation to all students in the country. Second, the unequal number of students across the individual years of study may limit the statistical processing of the collected data. In this case, however, these differences stem from admission quotas: the number of BSc students is three to four times higher than that of MSc students. Third, the quality of the results is limited by the use of research-developed 17 item question set. Although the questions were prepared in collaboration with linguists and PC specialists, they primarily address practical aspects of PC functioning in Poland. They serve as an additional verification of the responses provided to the translated PaCKS scale items.

The detailed section of the survey proved to be more challenging, as the questions were often practical in nature or required in-depth knowledge of PC. It is not possible to compare the responses to these questions with other studies, as they are original, researcher-developed items essentially intended to verify the quality and effectiveness of student education on this subject. It would be intriguing to see how students from other medical universities would respond to these same or similar detailed questions.

## 6 Conclusions

Previous studies underscored that insufficient training influences nurses’ PC knowledge. And although the results of our study were relatively satisfactory, it becomes evident that nursing students require greater clarity and understanding of the nuances of palliative care. Therefore, it is essential to implement action plans to address this issue, especially given the rise in chronic and debilitating diseases, including neurological disorders leading to dementia. It is recommended to integrate PC into nursing curricula not only at the BSc level but also within MSc programmes. Courses should address both basic and advanced aspects of PC, although for MSc students they should additionally include elements of palliative care management in various clinical situations. Addressing this knowledge gap could be achieved through comprehensive university education that covers palliative-, hospice- and long-term care, clarifying the appropriate contexts for each. However, it should be noted that our study concerned the knowledge of students from only one medical university. Thus, in addition to achieving the above educational goals, due to the surprising results of some of our observations, it is advisable to conduct comparative research using an identical or similar tool to assess students’ knowledge of PC - in Poland and other countries.

In summary, the students participating in this study, representing various levels of education at a medical university, demonstrated overall good knowledge of PC as measured by the PaCKS scale. Naturally, the distribution of correct answers indicates substantial variation in knowledge levels among individual students. However, the mean scores obtained on this scale reflect favourably on the respondent group as a whole.

## Data Availability

The original contributions presented in the study are included in the article/Supplementary Material, further inquiries can be directed to the corresponding author.
